# Influence of Sulfododecenylsuccinylation on the Adhesion to Fibers and Film Properties of Corn Starch for Warp Sizing

**DOI:** 10.3390/polym15061495

**Published:** 2023-03-17

**Authors:** Chaohui Zhang, Wei Li, Zhenzhen Xu

**Affiliations:** Department of Textile Engineering, College of Textiles and Garments, Anhui Polytechnic University, Wuhu 241000, China

**Keywords:** corn starch, sulfododecenylsuccination, degree of substitution, adhesion, film properties

## Abstract

To improve the film brittleness and adhesion to fibers of dodecenylsuccinated starch (DSS), DSS samples were sulfonated with excess NaHSO_3_ to prepare a series of sulfododecenylsuccinated starch (SDSS) samples with different degrees of substitution (*DS*). Their adhesion to fibers, surface tensions, film tensile properties and crystallinities, and moisture regains were studied. The results showed that the SDSS was superior to DSS and acid-thinned starch (ATS) in the adhesion to cotton and polyester fibers and breaking elongation of film but was inferior to them in tensile strength and degree of crystallinity of film, which revealed that sulfododecenylsuccination could further improve the adhesion of ATS to both fibers and reduce its film brittleness compared to starch dodecenylsuccination. With the increase in the *DS*, the adhesion to both fibers and the film elongation of SDSS gradually increased and then decreased, while its film strength consistently decreased. Considering adhesion and film properties, the SDSS samples with a *DS* range of 0.024-0.030 were recommended.

## 1. Introduction

Nowadays, the rapid development of the world economy has brought about increasingly serious environmental pollution, which means starch receives increasing attention and has been widely used as a sizing agent in the textile field due to its low cost, environmental friendliness [1], renewability [2], biodegradability [3] and so forth. However, the massive cyclic structure of glucose residues and hydroxyl groups in the residues make starch molecules have strong intermolecular forces, resulting in the brittle and rigid features of starch. These features are the main reasons that starch presents the inherent shortcomings of strong film brittleness and insufficient adhesion to fibers [4]. Sufficient adhesion to fibers has been described as a valuable behavior for the use of starch in the textile sizing field [5]. In this field, sufficient adhesion is extremely important since adhesion can not only promote yarn strength but also reduce the amount of yarn hairs lost by cementing them back onto the yarn body [6]. Consequently, adhesion has been known as a considerably important index for evaluating the quality of starch-based sizing agents. Moreover, the film covered around yarn surfaces can provide important protection from mechanical abrasions for the yarn, and thus accelerate weaving [7]. Therefore, starch film must have a low brittleness to protect the yarn from abrasive actions to ensure good quality of fibrous products. As a result, lowering the film brittleness of starch and promoting its adhesion to fibers is considerably important to improve its application in the textile sizing field.

Fortunately, the application of starch can be improved by different modification methods [8], such as starch dodecenylsuccinylation. This process occurs when dodecenylsuccinylated starch (DSS) as an organic modified starch is prepared through the reaction of starch with dodecenylsuccinic anhydride (DDSA) in alkaline conditions. It has been pointed out that the introduction of dodecenylsuccinate substituents onto starch can reduce its film brittleness and improve its adhesion to fibers [9]. The dodecenylsuccinate substituents have high space volumes for producing strong steric hindrance to the formation of hydrogen bonds between starch hydroxyls and the regular arrangement between starch chains, imparting a strong plasticization to starch film (formed around fiber surfaces from starch paste) or starch adhesive layers (formed between fibers from starch paste). In addition, the dodecenylsuccinate substituents introduce a hydrophobic element in the form of dodecenyl groups onto starch, which can combine normally hydrophilic starch to make all starch molecules present with an amphiphilic feature, subsequently imparting a high surface activity to the derivative [10]. Therefore, when the substituents are introduced with a low percentage content, the starch retains a strong hydrophilicity. Introducing the dodecenylsuccinate groups can reduce the surface tension of starch paste and improve the wetting and spreading of the paste on fiber surfaces, facilitating the adhesion of starch to fibers. However, when the percentage content is higher, the higher hydrophobicity generated by the hydrophobic dodecenylsuccinate substituents will severely reduce the water-dispersibility of starch. It has been confirmed that the lowered water-dispersibility is unfavorable to the wetting and spreading of starch aqueous paste, thereby producing an adverse effect on adhesion [9]. Therefore, it is necessary to increase the hydrophilicity of DSS for overcoming the adverse effect on adhesion. The available measure is to conduct a sulfonation of DSS with excessive NaHSO_3_, by which a new modified starch (sulfododecylsuccinated starch, SDSS) containing hydrophilic sulfonates and hydrophobic dodecenylsuccinates can be obtained. The hydrophilic sulfonates may exert a positive effect on the improvement of water-dispersibility of DSS as well as the increase in the moisture content of DSS film or the adhesive layer. Moisture can be considered an important plasticizer of starch [11,12]. Therefore, a further reduced film brittleness and improved adhesion to fibers may be expected.

Nowadays, there is no study about the effect of further sulfonation on the adhesion and film properties of DSS, and no work confirms that sulfonation can further improve the properties of starch. Accordingly, one aim of this study is to reveal if sulfonation can further improve these properties. Another aim is to determine a suitable sulfododecylsuccination level since the properties of modified starch commonly depend on the modification level. Therefore, a series of DSS and SDSS samples were prepared and characterized with Fourier Transform Infrared (FTIR). Surface tension and adhesion to polyester and cotton fibers of their pastes were determined. Furthermore, their films were characterized using X-ray diffraction (XRD), and film properties such as tensile property and moisture regain were also estimated. This study will lay a certain technical foundation for the use of SDSS as a new adhesive in warp sizing.

## 2. Experimental Section

### 2.1. Materials

Raw corn starch with an apparent viscosity of 50 mPa·s (measured at 6 wt% and 95 °C for 1 h) was purchased from Yixing Starch Factory (Yixing, Jiangsu Province, China). The reagents such as NaOH, HCl, NaHSO_3_ and Na_2_CO_3_ were of analytical grades and purchased from Nanjing Chemical Reagent Co., Ltd. (Nanjing, Jiangsu Province, China). The chemical grade reagent (dodecenylsuccinic anhydride, DDSA) was supplied by Hangzhou Zhongxiang Chemical Co., Ltd. (Hangzhou, Zhejiang Province, China). Pure polyester roving (365 tex) and cotton roving (375 tex) utilized as substrates to test the adhesion were purchased from Shandong Huawei Textile Co., Ltd. (Rizhao, Shandong Province, China).

### 2.2. Sulfododecenylsuccinylation of Starch

Due to the large apparent viscosity, the raw starch was first treated with HCl solution according to the method in the literature [13] to obtain an acid-thinned starch (ATS) sample that had an apparent viscosity of 10 mPa·s.

**Scheme 1:**
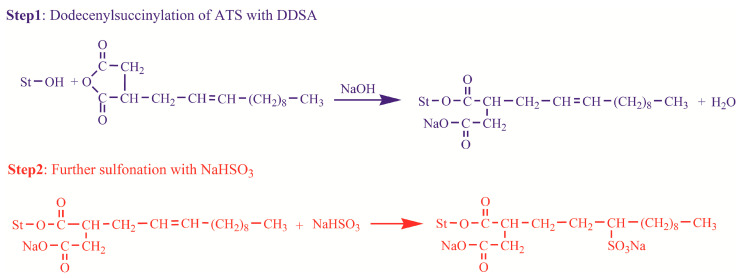
The dodecenylsuccinylation of ATS with DDSA and subsequent sulfonation with NaHSO_3_ for preparing SDSS sample.

Sulfododecenylsuccinylation of starch includes two steps, i.e., dodecenylsuccinylation of ATS with DDSA and subsequent sulfonation with NaHSO_3_, as shown in Scheme 1. Dodecenylsuccinylation of ATS with DDSA was conducted as follows: Dried ATS was mixed with distilled water to prepare a starch suspension with a mass fraction of 40%. The suspension was adjusted to pH 8.5–9.0 by a NaOH solution (3 wt%) and heated to 40 °C under mechanical stirring. Then, DDSA (its amount is indicated in Table 1) ethanol solution with a volume fraction of 20% was slowly added with a separating funnel; meanwhile, the pH of the suspension was maintained at 8.5–9.0 with the NaOH solution. After the addition, the reaction was continued for 4 h, and then the raw product was neutralized to pH 6.5–7.0 with 3 wt% HCl solution. Finally, the product was filtered, washed with ethanol-water solution, dried in an oven and pulverized to obtain a powdered DSS sample. By adjusting the mass percentage of DDSA to ATS, DSS samples with different degrees of substitution were prepared.

The prepared DSS was further sulfonated with NaHSO_3_ to prepare the SDSS. Briefly, dried DSS was mixed with distilled water to prepare a 40 wt% suspension, which was heated to 45 °C under mechanical stirring. Then, the NaHSO_3_ (fivefold the weight of dodecenylsuccinates introduced onto starch) was added into the suspension, and the reaction was maintained for 5 h. The suspension was adjusted to pH 6.5–7.0 with Na_2_CO_3_ solution (1 mol/L), and followed by filtering, washing with ethanol-water solution, oven-drying and pulverizing to obtain a granular SDSS sample.

### 2.3. Fourier Transform Infrared Analysis

Granular ATS and SDSS samples were analyzed on a Nicolet Nexus 470 Fourier Transform Infrared (FTIR) Spectrophotometer (Thermo Electron Corporation, Waltham, MA, USA), and their spectra were collected to reveal if the dodecenylsuccinates and sulfododecenylsuccinates had been successfully introduced onto starch chains. For each spectrum, the wavenumber ranged from 500 cm^−1^ to 3000 cm^−1^.

### 2.4. Determination of DS and Sulfonation Efficiency

*DS* of SDSS can be measured by titrating the content of double bonds in the samples before and after sulfonation according to the work in [14] because the double bonds in the DSS chains cannot be completely converted into single bonds during the sulfonation reaction. *DS*_2_ of SDSS and sulfonation efficiency (*R*, the percentage of the sulfonated double bonds) were calculated with the following equations:

(1) $$X_{1}=\frac{(V_{1}-V_{2})\times C\times 0.0799}{W}\times 100\%$$

(2) $$DS_{1}=\frac{162X_{1}}{79.904\times \left(2-3.329X_{1}\right)}$$

(3) $$R=\frac{X_{1}-X_{2}}{X_{1}}\times 100\%$$

(4) $$DS_{2}=DS_{1}\times R$$

where *X*_1_ is the bromine value of DSS, *V*_1_ and *V*_2_ are the volumes (mL) of the Na_2_S_2_O_3_ standard solution required for the titration of DSS and ATS, respectively, *C* is the molar concentration (mol/L) of the Na_2_S_2_O_3_ standard solution, *W* is the dry mass (g) of DSS, *DS*_1_ is the degree of substitution of DSS, and *X*_2_ is the bromine value of SDSS.

### 2.5. Measurement of Surface Tension

The surface tension of the paste was determined on a DCAT 21 Automatic Tensiometer (Dataphysics Co. Ltd., 70794 Filderstadt, Germany) [15]. The remaining paste (200 mL) prepared during the measurement of the bonding force was firstly cooled to room temperature and then the measurement of its surface tension was carried out in duplicate at room temperature. In addition, the mean value of two independent measurements was presented.

### 2.6. Preparation and Measurement of Starch Films

The cooked starch paste was cast onto a smooth polyester film (650 mm in length and 400 mm in width) at 65% relative humidity (RH) and a temperature of 20 °C and dried in order to form the starch film according to our previous study [15].

Before the measurement, the film prepared was cut into 200 mm × 10 mm strips and stored at 20 °C and 65% RH for 24 h. Their thicknesses were tested with a YG141 Thickness Gauge (Changzhou Textile Electronic Tester Co., Ltd., Changzhou, Jiangsu, China), and then their tensile strength and elongation at break were measured on a YG026D Electronic Strength Tester (Ningbo Textile Instrument Factory, Ningbo, Zhejiang, China) according to the ASTM D 882–02 method. For each case, the data for tensile strength and elongation at break reported were the mean values of twenty successful measurements.

Moisture regain (*R*_m_, %) adopted to evaluate the film hygroscopicity was measured by comparing the difference between the initial mass of the film (stored at 20 °C and 65% RH for 24 h) to its constant mass after being dried at 105 °C over a given period, as reported in our previous work [16]. The film that had been stored at 65% RH and 20 °C for 24 h was cut into square pieces of about 1 cm × 1 cm which were exactly weighed (denoted by *m*_1_). Afterwards, the pieces were dried to constant masses in an oven at 105 °C and exactly weighed again (denoted by *m*_2_). Moisture regain was measured in duplicate and calculated with the following Equation (5):

(5) $$R_{m}=\frac{m_{1}-m_{2}}{m_{2}}\times 100\%$$

The XRD patterns of ATS, DSS and SDSS films were collected on an XRD−6000 X-ray Diffractometer (Shimadzu Co., Nakagyo-ku, Kyoto 604-8511, Japan) equipped with a wavelength of 0.154 nm monochromatic CuKa radiation at 40 kV and 30 mA. The diffraction angle (2*θ*) was in an angular range of 5° to 60° with a speed of 4°/min and a 0.02° angular step size.

### 2.7. Measurement of Bonding Force

The adhesion of DSS, SDSS or ATS to polyester and cotton fibers was investigated via measuring the bonding force of slightly sized roving according to a standard method (FZ/T 15001–2008) in China. The measurement comprises three main steps: (a) preparing a starch paste, (b) collecting the sized roving by immersing the roving into the paste and air-drying, and (c) conducting a tensile test [15]. Firstly, a 1 wt% DSS, SDSS or ATS aqueous paste (800 mL) was prepared by heating the DSS, SDSS or ATS aqueous dispersion to 95 °C and keeping it at this temperature for 1 h under consistent agitation. Then, the paste (600 mL) was applied for sizing the polyester or cotton roving that had been wound carefully onto a rectangular steel frame for 5 min. After the paste on the roving had been air-dried, the roving samples were collected and equilibrated at 65% RH and 20 °C for 24 h. Afterwards, the bonding forces of the roving samples were obtained by conducting the drawing tests on a YG026D Electronic Strength Tester (Ningbo Textile Instrument Factory, Ningbo, Zhejiang, China) at a cross-head speed of 50 mm/min with an initial chuck distance of 100 mm. For each case, the data reported were the average of the bonding forces and the coefficient of variation.

### 2.8. Statistical Analysis

Statistical significance was performed with one-way analysis of variance using the ORIGIN 6.0 (OriginLab Inc., Northampton, MA, USA) for Windows program. The data presented were considered significantly different at *p* < 0.05.

## 3. Results and Discussion

### 3.1. Characterization Analysis

**Figure 1:**
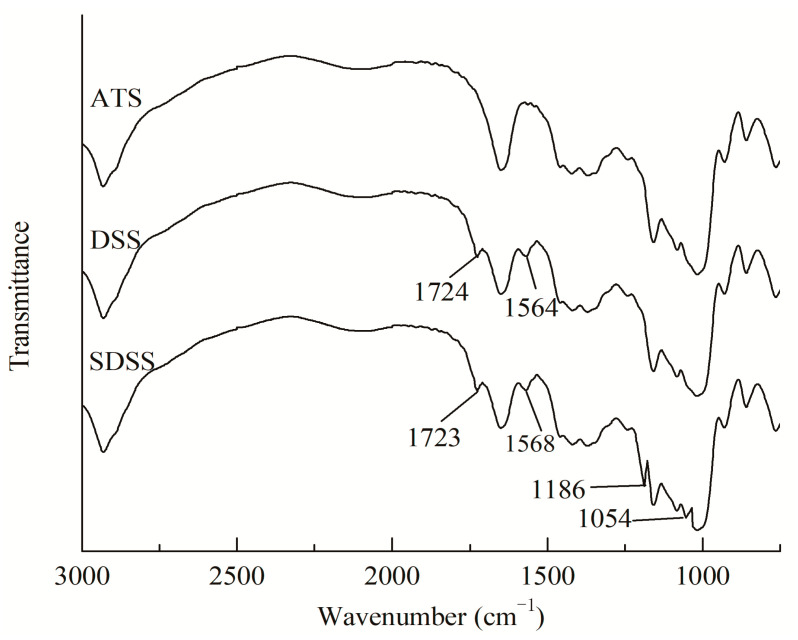
FTIR spectra of ATS, DSS and SDSS.

The successful introduction of dodecenylsuccinates and sulfododecenylsuccinates into starch molecules was demonstrated by FTIR spectra of SDSS, DSS and ATS, as displayed in Figure 1. The peaks at the wavenumbers 2931 cm^−1^ in the spectrum of ATS and 2930 cm^−1^ in the spectra of DSS and SDSS corresponded to the asymmetric stretching vibration of C–H [17]. Compared with the infrared spectrum of ATS, the spectra of DSS and SDSS presented new absorption peaks at 1724 cm^−1^ and 1723 cm^−1^, respectively, which corresponded to the stretching vibration of ester carbonyl groups [18]. The spectra also exhibited new absorption peaks at 1564 cm^−1^ and 1568 cm^−1^, respectively, which indicated the asymmetric stretching vibration of carboxylates [19], demonstrating the successful introduction of dodecenylsuccinates in the DSS and SDSS molecules. In addition, compared with the infrared spectra of DSS and ATS, the FTIR spectrum of SDSS showed two new peaks at the wavenumbers 1186 cm^−1^ and 1054 cm^−1^, which corresponded to the symmetric stretching vibration of sulfonic ions [20]. This observation indicated the presence of sulfonates in the SDSS molecules and confirmed the successful introduction of sulfododecenylsuccinates into the starch molecules.

The *DS*_1_ values of DSS samples, *DS*_2_ values of SDSS samples and sulfonation efficiencies (percentage of the double bonds in the dodecenylsuccinate substituents sulfonated) were measured and illustrated in Table 1. As found, the *DS*_1_ values were dependent on the dry masses of the DDSA used in the esterification, i.e., as the masses of DDSA were added from 0 to 96 g, the DS_1_ values displayed a gradual increase from 0 to 0.041, which indicated that DSS with an esterification level range from 0 to 0.041 could be acquired by the reaction of ATS with DDSA in an aqueous medium. Additionally, sulfonation efficiencies were higher than 90%, which indicated that the percentages of sulfonated double bonds in the dodecenylsuccinate substituents all exceeded 90%. This observation implied that the efficiencies were less dependent on the extent of starch dodecenylsuccination. Accordingly, as the efficiencies were all over 90% and the *DS*_1_ gradually increased from 0.008 to 0.041, the *DS*_2_ of SDSS samples also consistently increased from 0.0078 to 0.038.

### 3.2. Influence of Starch Sulfododecenylsuccination

#### 3.2.1. Influence on Film Properties

The tensile properties (tensile strength and breaking elongation) of DSS and SDSS films are presented in Table 2.

Compared with the ATS film, the DSS and SDSS films showed a significant difference in the breaking elongation, and the DSS and SDSS (DS ≤ 0.024) films had no significant difference in tensile strength (*p* < 0.05). As the DS values were in the range of 0.030–0.038, the SDSS films had a significant difference in tensile strength compared with ATS film (*p* < 0.05). This indicated that the sulfododecenylsuccination could not only obviously lessen the film brittleness of ATS but also further lessen the film brittleness of starch compared with dodecenylsuccination. In addition, the tensile strength and breaking elongation of SDSS films were in correlation with the DS. As the DS increased, the elongation consistently increased, and the elongation of SDSS (0.024–0.038) films had a significant difference with that of SDSS (0.0078) film, whereas the strengths gradually decreased and had no significant difference with each other (*p* < 0.05). The maximal elongation (3.65%) of the SDSS film was observed at the DS of 0.030 and then reduced. Accordingly, the SDSS (DS = 0.030) film with a higher elongation is preferable to the DSS (DS = 0.032) and ATS films for application in warp sizing.

**Figure 2:**
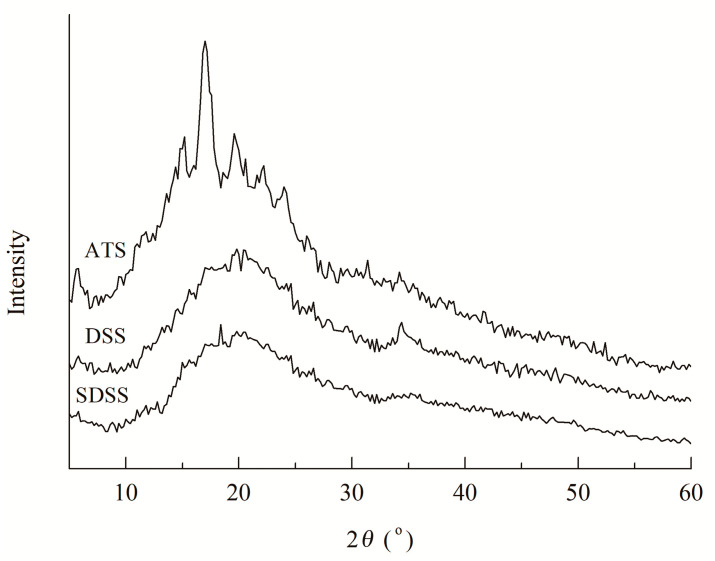
X-ray diffraction of ATS, DSS and SDSS films.

During the film-forming process of starch paste, the linear amyloses in the paste can create regular arrangements due to the hydrogen bonding between hydroxyls, and then a three-dimensional network structure is formed [21]. It is the main reason why starch film presents great brittleness and low deformation [22]. After the introduction of dodecenylsuccinate and sulfododecenylsuccinate substituents, the parallel arrangements can be disturbed through their steric hindrance, impeding the growth of crystals on the nucleus during film formation. As a result, a lowered degree of crystallinity may be expected by the introduction of the substituents. The degree of crystallinity can be analyzed using the XRD technique, as displayed in Figure 2. Compared with the XRD pattern of ATS film, it could be seen that the intensities of the peaks for DSS and SDSS films were much lower than those for the ATS film. The lower intensities indicated that both substituents lessened the formation of crystalline structures in the starch film; consequently, the DSS and SDSS films had lower crystallinities (15.7 and 12.8%) than the ATS film (21.7%). Therefore, the SDSS film had the highest elongation and lowest strength, and the ATS film had the lowest elongation and highest strength, which indicated that the sulfododecenylsuccination could subsequently diminish film brittleness and increase its extensibility in comparison with the dodecenylsuccination.

**Figure 3:**
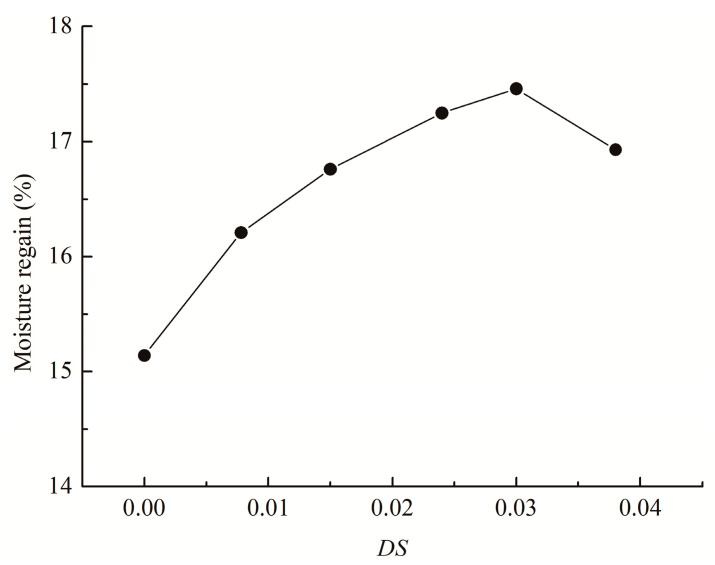
Influence of sulfododecenylsuccination on the moisture regain of starch film.

As is well known, water can be described as a good plasticizer of starch film [23,24]. Therefore, the influence of dodecenylsuccination and sulfododecenylsuccination on the moisture regain of ATS film was estimated, and the results are shown in Figure 3. It could be found that both chemical modifications promoted the moisture regain of ATS (*DS* = 0) film. With the increases in sulfododecenylsuccination levels (*DS*_2_) from 0.0078 to 0.030, the regains gradually increased from 16.2% to 17.5% and then showed a certain decrease as the level reached 0.038. Additionally, the regains of DSS (*DS*_1_ = 0.008 and 0.032) films were 15.9% and 17.1%, respectively, and that of the ATS film was 15.1%. This indicated that sulfonation could further increase the moisture regain of starch film. Undoubtedly, the increased regain can absorb more water and store it in the film, which will provide a higher plasticization for the film, thereby favoring the increase in elongation as well as the reduction in strength and generating a diminished film brittleness.

#### 3.2.2. Influence on Adhesion

The influence of sulfododecenylsuccination on the adhesion of ATS to polyester and cotton fibers was evaluated, as shown in Table 3. 

It could be found that the bonding forces of ATS (*DS* = 0) to both fibers were 103.3 N and 54.7 N, respectively, and DSS was superior to ATS in the bonding forces to both fibers. The forces of SDSS samples to both fibers were related to their *DS* values. With the rises in *DS* values, the forces were gradually increased, and when the value was 0.024, the forces reached their maximum values of 125.9 N for polyester fibers and 64.6 N for cotton fibers. When the DS of DSS was 0.032, its bonding force to polyester fibers was significantly different with that of ATS (*p* < 0.05), but the force to cotton fibers was insignificantly different with that of ATS (*p* < 0.05); however, the SDSS samples with a DS range of 0.0078–0.038 presented a significant difference with ATS in the force on polyester fibers (*p* < 0.05), and the SDSS samples with a DS range of 0.015–0.032 had a significant difference with ATS in the force on cotton fibers (*p* < 0.05). These observations indicated that sulfododecenylsuccination could further improve the adhesion of ATS to both fibers in comparison with dodecenylsuccination. Based on these results, the suitable sulfododecenylsuccination level, i.e., *DS* of SDSS, for promoting the adhesion of ATS to both fibers was 0.024 in the DS range of 0.0078 to 0.038.

Starch, as one of the most globally important biopolymers in the world [25], is produced mainly using plants [26]. Structurally, gelatinized starch paste is a suspension of the granular fragments of amylopectin dispersed in a continuous phase that consists of dissolved amylose [27]. Amylose molecules in the continuous phase have the inclination to parallel arrangements and create hydrogen bonding between the hydroxyl groups in molecules, thereby forming insoluble aggregates. These insoluble aggregates can deteriorate the water-dispersibility of starch, which negatively affects the wetting and spreading of starch paste on fiber surfaces and leads to a low adhesion to fibers. However, dodecenylsuccinates and sulfododecenylsuccinates introduced onto starch molecules can produce strong steric hindrance to parallel arrangements and hydrogen bonding, thereby reducing the formation of insoluble aggregates. In addition, hydrophilic carboxylates and sulfonates can increase the hydrophilicity of starch. These favor the improvement of water-dispersibility in starch, producing a positive influence on the wetting and spreading, and thus improving the adhesion of starch to fibers. Compared with dodecenylsuccinates, sulfododecenylsuccinates (dodecenylsuccinates + sulfonates) will generate stronger steric hindrance and hydrophilicity. Consequently, a greater improvement in adhesion may be expected.

**Figure 4:**
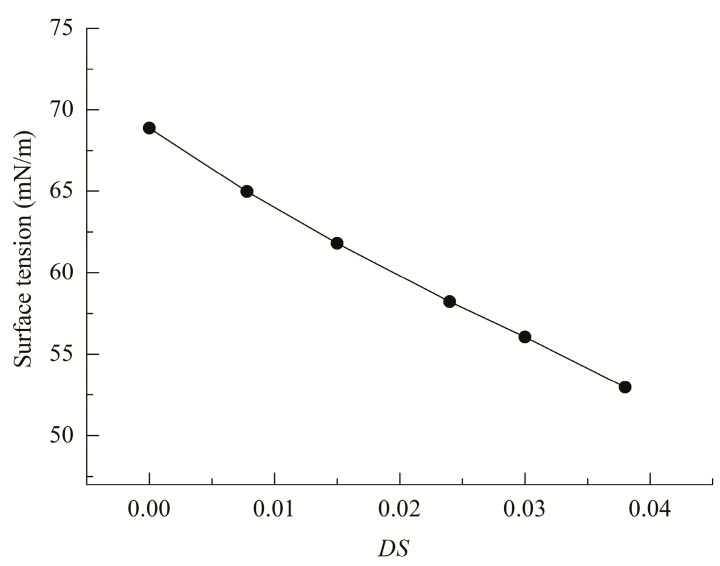
Influence of the sulfododecenylsuccinate substituents on the surface tension of starch paste.

Moreover, there are hydrophobic ester groups and hydrophilic groups in dodecenylsuccinate or sulfododecenylsuccinate substituents, which impart the substituents with a biparental structure similar to surfactant. Therefore, introducing these substituents will favor a decrease in the surface tension of starch paste. The surface tension of SDSS paste was determined and presented in Figure 4. As seen, the surface tensions of SDSS pastes were lower than that (68.9 mN/m, *DS* = 0) of ATS paste. Additionally, the tensions of DSS (*DS* = 0.008 and 0.032) pastes were also determined and the results were 65.9 mN/m and 59.4 mN/m, respectively. These results showed that introducing the dodecenylsuccinate or sulfododecenylsuccinate substituents really reduced the surface tension of ATS paste. The rises in the DS values presented a gradually reduced surface tension for the SDSS paste. It has been concluded that a reduced surface tension commonly provides a positive effect on the wetting and spreading of starch paste on fiber surfaces, and thus improving the adhesion to fibers [28]. This is also one main reason that the introduction of dodecenylsuccinate or sulfododecenylsuccinate substituents favors the improvement of adhesion.

Due to the brittle behavior of starch, during the drying process of starch paste that existed between fibers, strong internal stresses can be induced within the starch adhesive layer and at the interface between the layer and fibers with the volume shrinkage of the paste. It has been known that these stresses can damage adhesion [13]. Obviously, the internal plasticization generated by the dodecenylsuccinate or sulfododecenylsuccinate substituents and the external plasticization arisen from the moisture absorbed by the hydrophilic groups in the substituents can exert an important effect on diminishing the brittle behavior of starch, thereby favoring the reduction in stresses, and thus increasing the adhesion to both fibers. For polyester fibers, the ester groups in the substituents and polyester chains have a structural similarity which can increase the van der Waals force at the interfaces between starch layers and polyester fibers to reinforce interfacial actions. For cotton fibers, the carboxyl groups or sulfonates introduced onto starch raise the polarity of starch and promote intermolecular forces on the starch–cotton interface. The variation is favorable to the promotion of adhesion to cotton fibers. It is also responsible for the improvement in adhesion after starch dodecenylsuccination or sulfododecenylsuccination.

Furthermore, with the increase in the DS of SDSS, the introduction of more hydrophobic dodecenylsuccinates will obviously reduce the water-dispersibility of starch in an aqueous medium as well as the moisture absorption of starch adhesive layers, which will lead to a negative impact on adhesion. Therefore, when the DS of SDSS exceeds 0.024, its adhesion to both fibers gradually reduces.

## 4. Conclusions

Based on previous results, it could be concluded that the sulfonation of DSS was an efficient method of further promoting the properties of ATS. After the further sulfonation of DSS, i.e., sulfododecenylsuccination of ATS, the sulfododecenylsuccinates were successfully introduced onto ATS molecules, which was confirmed using the FTIR technique. Compared with dodecenylsuccinates, introducing sulfododecenylsuccinates onto the ATS molecules could not only further promote the adhesion of ATS to cotton and polyester fibers but also further reduce the film brittleness (present increased film elongation and reduced film strength) of ATS. Compared with dodecenylsuccinates, sulfododecenylsuccinates provide stronger steric hindrance and higher water absorption, thereby exerting a greater plasticization to starch film and subsequently reducing film brittleness. Plasticization can also reduce the stresses within starch adhesive layers and at the layer–fiber interface, and sulfododecenylsuccinates can facilitate the wetting and spreading of starch paste on fiber surfaces due to the lowered surface tension of starch paste, thereby further improving the adhesion to both fibers. Adhesion and film properties were dependent on *DS*. With the increase in the *DS* of SDSS, the breaking elongation of SDSS film increased and then decreased. When the *DS* was 0.030, it reached its maximum, and the film strength gradually decreased. As the *DS* rose, the adhesion of SDSS to polyester and cotton fibers increased, reached its maximum values when the *DS* was 0.024, and then decreased. Considering the tensile properties of film and adhesion, the SDSS with a *DS* range of 0.024–0.030 showed potential in applications to cotton and polyester sizing. This study will lay an important foundation for the use of SDSS as a new adhesive in the warp sizing field.

## Figures and Tables

**Table 1:** Characterization of starch sulfododecenylsuccination.

Dodecenylsuccination		Sulfonation		
Dry Mass of DDSA (g)	*DS* of DSS (*DS*_1_)	Dry Mass of NaHSO_3_ (g)	Sulfonation Efficiency (%)	*DS* of SDSS (*DS*_2_)
11	0.008	9.28	97.5	0.0078
24	0.016	18.37	93.8	0.015
43	0.025	28.39	96.0	0.024
63	0.032	36.01	93.8	0.030
96	0.041	45.64	92.7	0.038

Note: dry mass of ATS was 180 g, and dry mass of NaHSO_3_ was 5 times the mass of dodecenylsuccinates.

**Table 2:** Influence of sulfododecenylsuccination on tensile strength and breaking elongation of starch film.

Starches	DS	Breaking Elongation		Tensile Strength	
		Average (%)	CV (%)	Average (MPa)	CV (%)
ATS	/	2.1 a	8.14	28.6 a	7.85
DSS	0.008	2.5 b	9.26	27.6 ab	7.36
	0.032	3.3 c	8.35	25.1 ab	6.79
SDSS	0.0078	2.7 b	9.75	26.9 ab	6.97
	0.015	3.2b c	9.42	26.1 ab	7.51
	0.024	3.5 c	9.64	25.2 ab	7.76
	0.030	3.7 c	9.38	24.4 b	7.48
	0.038	3.3 c	9.17	24.2 b	6.70

Note: data points with a different small letter denote significant difference (*p* < 0.05), and the points containing the same small letter denote no significant difference (*p* < 0.05).

**Table 3:** Influence of sulfododecenylsuccination on the adhesion of starch to polyester and cotton fibers.

Starches	DS	Bonding Force to Polyester Fibers		Bonding Force to Cotton Fibers	
		Average/N	CV/%	Average/N	CV/%
ATS	/	103 a	6.9	55 a	6.0
DSS	0.008	110 ab	6.7	58 ac	6.0
	0.032	118 bc	6.5	61 ac	6.7
SDSS	0.0078	115 bc	6.0	60 ac	5.7
	0.015	121 bc	6.9	63 bc	5.9
	0.024	126 c	7.1	65 bc	6.4
	0.030	123 c	7.3	63 bc	6.8
	0.038	118 bc	7.6	61 ab	6.6

Note: data points with a different small letter denote significant difference (*p* < 0.05), and the points containing the same small letter denote no significant difference (*p* < 0.05).

## Data Availability

The raw/processed data required to reproduce these findings cannot be shared at this time as the data also forms part of an ongoing study.

## Abbreviations

Dodecenylsuccinated Starch	DSS
Sulfododecenylsuccinated Starch	SDSS
Degree of Substitution	DS
Acid-Thinned Starch	ATS
Dodecenylsuccinic Anhydride	DDSA
Fourier Transform Infrared	FTIR
X-ray Diffraction	XRD
Relative Humidity	RH
